# The Medical Department of the University of Michigan and Preliminary Education

**Published:** 1858-11

**Authors:** 


### EDITORIAL.

### THE MEDICAL DEPARTMENT OF THE UNIVERSITY OF MICHIGAN AND PRELIMINARY EDUCATION.

All who have been in the habit of looking over the Annual Announcements of the Medical College at Ann Arbor, Michigan, and its former organ, the *Peninsular Journal*, are aware that great credit has been claimed for that institution on account of a pretended compliance with the recommendations of the American Medical Association in relation to the length of lecture term and a standard of *preliminary education*. And the readers of this journal will remember, that two or three years since we were plunged into a protracted controversy with the senior editor of the *Peninsular Journal* for simply denominating some of these claims mere *bombastic pretences*. As one of the chief items for which they claimed credit for being in advance of all other schools, both in that controversy and elsewhere, was the adoption of a high standard of *preliminary education*, we take the liberty of placing before our readers the following report of the proceedings of a recent meeting of the Board of Regents of the State of Michigan, taken from a Detroit paper:

2½ O'CLOCK P.M.

Mr. McIntyre presented a report on the question of the removal of the Medical Department to Detroit. The report stated that the committee had investigated the matter, and found legal objection in the enabling act of the Legislature; that the University was to be located in or near the village of Ann Arbor; that it was in accordance with the real or implied contract with the grantors of College Square to the institution, on condition that the University, not a branch, but the whole University, should be there located; that the great want of medical students was not so much a course of clinical instruction, as a more thorough preparatory course; that the people of Michigan did not wish the removal; that nobody did wish it but one or two Detroit professors and the friends they influenced; that the Medical Department of the Michigan University, without extensive clinical instruction, has more students than Yale and Harvard Medical Schools combined, and more than twice as many as that at Richmond, Va.; that professors should be required to reside at Ann Arbor, at least during term time; that irregularity of lectures was chargeable to the non-residence of professors, especially Professor Gunn; that Professors Gunn and Pitcher had conducted their discussion in their medical journals on the Medical Department removal question, with ungentlemanly personalities; that Professor Gunn had used language injurious to the reputation of the institution; that the Medical Department could only be removed at great pecuniary sacrifice; and, finally, that personal interest and selfish motives of one or two professors are the sole cause of the present agitation of the question.

The committee reported the following resolutions:

1. That the Medical Department of the Michigan University ought not to be removed to Detroit or elsewhere, and that we trust that agitation of the subject by professors and others connected with the institution will henceforth cease, as we consider such agitation not only useless but detrimental to the best interests of the institution.

2. That each candidate for admission to the Medical Department shall furnish satisfactory evidence of good moral character to the President, and, if not a graduate of this or some other college, he shall bear an examination in natural philosophy, the elements of mathematical science, including geometry and algebra, and such an acquaintance with Latin as shall entitle him to admission to the freshman class of the class course of the University, to be certified by the proper professors of that course. This resolution shall take effect on first day of July next.

3. That from and after the expiration of the approaching medical term, all the Medical Professors shall be required to reside at or near Ann Arbor during the medical lecture term.

The report was accepted.

Mr. Bishop presented a minority report:

1. There was nothing in the acts of the Legislature forbidding the location of branches of the University out of Ann Arbor.

2. That a large city is best for a Medical College, and Detroit therefore is the best place for it in the State.

3. It is not the interest of the University to be kept all in one place.

4. That the pecuniary state of the University does not at present admit of removal, therefore concludes that the whole subject should be indefinitely postponed.

This report was also accepted.

On motion of Mr. Parsons, the committee was discharged.

Mr. Bishop moved the adoption of the first resolution of the majority report, and said that he disapproved of allusion to the

Professor of Surgery; that the discussions of Professors Pitcher and Gunn have been dignified by the notice of the Board; that the clinical course at Detroit did not amount to a straw; that the people of Detroit did not care a single straw about having the Medical Department there; and that the Detroit bar did not want the Law Department there.

Mr. Brown moved that the resolution be amended by striking out all after first clause.

Mr. Baxter said that the committee had not, as hinted, stepped out of their way to attack non-resident professors.

Mr. McIntyre could not sit still and be charged by gentlemen from Detroit with making personal attacks upon any professor. He said that Professor Gunn had not kept within bounds of proper discussion, but had assailed the College and Board of Regents, and deserved rebuke at the hands of the Board.

Mr. Bishop said the committee had no business to attack Professor Gunn in a report on the removal of the Medical Department.

Mr. Ferry said we could not stop public discussion by professors or anybody else.

Mr. Brown would not object to the character of the report, but thought it should be confined to subject matter.

Mr. Baxter thought it was high time that the Regents should express their opinion upon and rebuke such kind of discussion by professors of the institution.

Mr. Parsons considered the discussion on the amendment fashionable, and would accordingly make a few remarks. He thought the amendment was right. He did not like the course of professors. He thought they shouldn't be employed if they acted thus; yet he concluded that the committee had no jurisdiction in the matter.

Mr. Ferry thought such discussions beneath notice.

Mr. Baxter accepted Mr. Brown's amendment.

The resolution was adopted.

Mr. Baxter moved that the second resolution be adopted.

Mr. Bishop moved that the opinions of Drs. Sager and Palmer be asked on the subject.—Adopted.

Dr. Sager said the question had been discussed by the Faculty. It had been the former practice to have requirements before admission; that now they were required at graduation instead. If applied to candidates for admission, it would very much diminish the classes, and, in fact, the whole department.

Mr. McIntyre wished to direct the Doctor's attention to the fact that undergraduates would go out and practice.

Dr. Sager said it could not be helped. He thought it would be more important that students in medicine should have a knowledge of botany and zoology than Latin and mathematics.

Dr. Palmer did not think that Latin was absolutely necessary for medical men; that the popular mind was turning against classics. He thought the resolution would reduce the medical class to ten or twelve. The requirements had better be made in mathematics and science than in Latin.

Dr. Tappan inquired what he thought would be the effect of requiring medical students to be graduates of a literary college.

Dr. Palmer said it would reduce the Medical Department to nothing.

The resolution was tabled, on motion of Mr. McIntyre.

On motion of Mr Bishop, the third resolution was, after some discussion, tabled.

Dr. Palmer here wished to know if he, as a journalist, was required to refrain from subjects pertaining to the improvement of the profession?

Mr. McIntyre said no, nothing of the sort was thought of.

The Board adjourned till 8 o'clock p.m.

By reference to the second resolution appended to the report of Regent McIntyre, it will be seen that so far from having had any standard of preliminary education for admission into the medical department of the university, the committee have only just now proposed one for adoption. And what became of the proposition? The report says that Professors Palmer and Sager, on invitation, appeared before the Board, and so decidedly opposed its adoption, that it was laid on the table, where it still sleeps. Yes, the same senior editor of the *Peninsular Journal*, who assailed us so furiously for merely suggesting that their annual boast about being "so far in *advance*" of all other medical institutions in their standard of preliminary requirements was mere bombast, actually takes the lead in opposing a serious proposition to establish a positive and valuable requisition on the subject. It is an old saying that MURDER WILL OUT; and so will TRUTH, at least, occasionally. The Professors distinctly admit that no standard of *preliminary education* is required for *admission* into the medical department of the University of Michigan.

How long before the profession will be gravely told that this institution has complied with the recommendations of the American Medical Association, and that it is "FAR *in advance* of all other institutions" in this country?

